# Mapping protein glycosylation through oxocarbenium ion generation

**DOI:** 10.1038/s42004-020-00352-7

**Published:** 2020-08-07

**Authors:** Andrew J. Bissette

**Affiliations:** Communications Chemistry, https://nature.com/commschem

## Abstract

Mass spectrometry allows the pattern of glycosylation in proteins to be mapped, but can be limited by the lability of glycosidic bonds. Now, a method exploiting this lability allows for direct mapping of glycan moieties in glycoproteins.

Proteins are often decorated with carbohydrates, and mapping these glycosylation patterns is vital for understanding their biological functions. Mass spectrometry offers powerful methods for doing so, but selectively fragmenting peptide bonds while retaining the labile glycosidic bonds found in these important post-translational modifications—and hence retaining information about the sites on the protein where they are located—poses a key analytical problem. Now, a team led by Jennifer Brodbelt from the University of Texas at Austin and David Vocadlo from Simon Fraser University report a method that exploits the intrinsic reactivity of carbohydrates to selectively locate glycosylation sites (10.1021/jacs.0c04710)^1^.

Serine and threonine residues are commonly functionalised with *O*-linked *N*-acetylglucosamine (O-GlcNAc) moieties, which serve to regulate a range of functions in the cell including stress responses. Several mass spectrometry methods can map the locations of O-GlcNAc moieties, and involve fragmenting the main backbone of the peptide and assigning a series of overlapping fragment ions. But collisional activation-based methods share a common drawback: the glycosidic bond is more easily fragmented than the peptide bonds in the protein backbone, in part because of neighbouring group participation from the *N*-acetyl group. Consequently, information about the original glycosylation sites can be lost. While a range of sophisticated methods targeting O-GlcNAc modifications exist, there remains a need for general and rapid methods that allow mapping of labile post-translational modifications.

Prof. Brodbelt and colleagues address this by exploiting, rather than resisting, the predisposed chemistry of O-GlcNAc fragments^1^. Rather than trying to avoid cleavage of the O-glycosidic bonds that link the carbohydrate to the peptide backbone, they target it deliberately using collisionally activated dissociation (CAD) mass spectrometry. “Our method takes advantage of the abundant glycan-specific ions, called oxonium ions, to automatically trigger a second MS/MS method called ultraviolet photodissociation (UVPD)”, explains Brodbelt. UVPD targets confirmed glycopeptides with intact glycosidic bonds and fragments the backbone, allowing mapping of the glycosylation pattern. In effect, CAD exploits the lability of glycosidic bonds by flagging glycosylated species for photochemical fragmentation and mapping (Fig. 1).

**Fig. 1:**
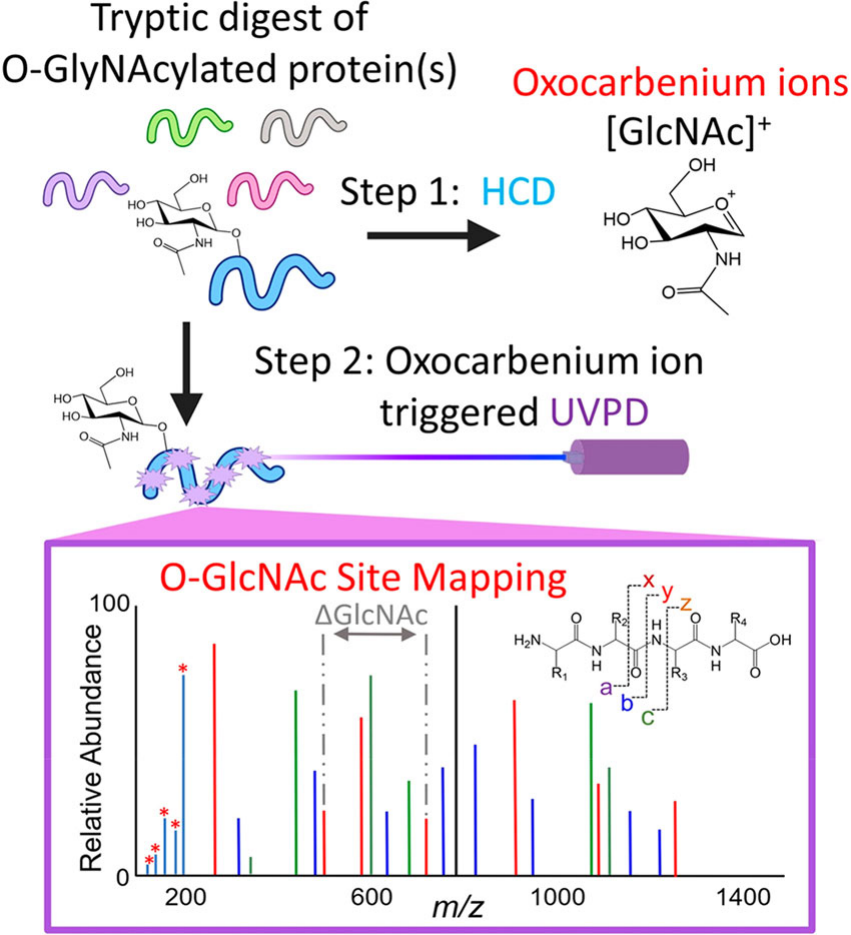
Mapping glycosylation oxocarbenium ion generation. Glycopeptides are identified using higher energy collisional dissociation (HCD), which generates reactive oxocarbenium ions. This triggers a second mass spectrometry method, ultraviolet photodissociation (UVPD), which allows mapping of glycosylation sites. Reprinted with permission from ref. ^1^. Copyright 2020 American Chemical Society.

The researchers suggest that the method may serve to complement other approaches to mapping O-GlcNAc. While both CAD and UVPD are known methods, critically, the combination of both may allow low-abundance glycoproteins to be analysed in the presence of higher concentrations of non-glycosylated proteins. By combining this approach to selecting and mapping glycosylated proteins with existing methods for enriching them from mixtures, direct insight into the functionalisation patterns of glycoproteins may be achievable even in complex mixtures. “Capitalizing on efficient fractionation and enrichment strategies would allow more successful analysis of whole cell glycoproteomes”, says Brodbelt, “especially with a focus on the understudied and challenging mapping of O-glycosylation”.

## References

[1] Escobar EE (2020). J. Am. Chem. Soc..

